# One Health research ethics review processes in African countries: Challenges and opportunities

**DOI:** 10.1016/j.onehlt.2024.100716

**Published:** 2024-03-22

**Authors:** Kuastros M. Belaynehe, Joseph M. Nguta, Melanie Lopez, Jinjian Mu, Dónal O'Mathúna, Getnet Yimer, Andréia G. Arruda

**Affiliations:** a Animal Health Institute (AHI), Epidemiology Unit, Sebeta, Ethiopia; b Department of Public Health, Pharmacology and Toxicology, Faculty of Veterinary Medicine, University of Nairobi, Kenya; c Boston Public Health Commission, Boston, MA, USA; d College of Nursing, The Ohio State University, Columbus, OH, USA; e Center for Global Genomics and Health Equity, Department of Genetics, Perelman School of Medicine, University of Pennsylvania, Philadelphia, PA, USA; f College of Veterinary Medicine, The Ohio State University, Columbus, OH, USA

**Keywords:** African countries, One Health research, Regulatory body, Research ethics, Research ethics committee

## Abstract

One Health research has gained attention over the past few decades due to its potential to improve health challenges across the globe. However, obtaining ethics approvals for timely implementation of One Health research is a challenge in some contexts. Our study was undertaken to describe various challenges faced by researchers, research ethics committees (RECs) and members of regulatory bodies in Africa. An online survey was conducted between March and June 2021. The effect of predictors, including respondents' role (e.g., REC member, regulator and/or One Health researcher), sex, education, age, and country, on the perception of challenges and opportunities when conducting and reviewing One Health research, was investigated using multivariable linear regression models. Participants with different roles did not perceive any of the examined challenges differently during review of One Health-related research; but female participants (*p* = 0.026) and those with ten or more years of experience (*p* = 0.0325) perceived insufficient One Health knowledge as less of a challenge. Professional role was an important predictor (*p* = 0.025) for the perception of the establishment of a mandatory One Health review system. Respondents with multiple roles perceived the creation of ad hoc committees for review of One Health research under emergency situations to be less important (p = 0.02); and REC members perceived the creation of such committees to be less feasible (*p* = 0.0697). Our study showed that perceptions of the importance and feasibility of opportunities for improvement of One Health research ethics review under emergency and non-emergency situations varied across professional roles. This emphasizes the need to consider such improvement strategies; and the need for continuous and timely evaluation for improvement of ethics review of One Health and emergency research in Africa.

## Highlights

• Timely and appropriate review of One Health and emergency research projects are critical.
• Training is vital for ethical decision-making skills of One Health actors in Africa.
• Interdisciplinary One Health research ethics review committees sought and feasible.
• Guidelines and SOPs are needed for One Health research ethics review system.
• Further bioethical investigation require to elucidate issues in One Health research.

## 1 Introduction

One Health recognizes the complex interconnectedness of human, animal, plant, microbial and environmental health, and responds with an interdisciplinary approach that aims to build capacity and resilience through preparation, responsiveness and mitigation during disease outbreaks [1]. The overall goal of One Health research is to improve the health of humans, animals, and ecosystems through capacity building, strengthening of local, regional, and global networks, and provision of evidence-based policy advice [2,3]. The One Health approach utilizes expert knowledge of researchers, public health practitioners, and other occupations within multiple disciplines starting from the local level and scaling to a global collaboration to achieve desired health goals [4]. This approach is suitable for sub-Saharan Africa as most of the thematic areas of relevance and research focus are linked to health, food security, and maintenance of livelihoods [5]. These important topics also include the prevention and control of neglected tropical zoonotic diseases and emerging infectious diseases, promotion of food safety, and prevention and control of antimicrobial resistance [5,6]. As such, the One Health approach can facilitate cross-sectoral, cross-disciplinary engagement and lead to improved economic outcomes.

One Health has quickly moved from a concept to a movement, with many projects, initiatives and platforms proliferating across sub-Saharan Africa implementing interdisciplinary problem-solving approaches to utilize the necessary policy and legal instruments to support community concerns [7,8]. This has simultaneously increased the demand for operational ethics review structures that efficiently serve the growing research environment and community [9]. At present, comprehensive information about challenges in One Health research ethics review processes at a global level is lacking. Such information would guide initiation of strategic interventions to improve decision making with regards to ethical aspects of One Health research, policy and guideline development. Such information from African countries could address major challenges in One Health research ethics review processes and regulations in Africa [10].

A well-established and well-resourced research ethics system is important to reduce the occurrence of unethical research practices. Emergency situations, epidemics, and disasters occur frequently, and when they do, rapid activation of emergency responses is of outmost importance to reduce potential damages from the epidemic or disaster. However, for the responses to be effective, coordinated, and appropriate, they need to be supported by relevant evidence that often can be acquired only by conducting research during the actual epidemic or emergency [11,12]. Emergency situations may require an extra level of effort from the researchers for their research-related procedures to accommodate concerns related to the emergency, or to effectively address ethical, legal, and social issues. Decision-making during public health emergency research requires that decision-making is timely and effective for the delivery of adaptive responses and effective interventions. These responses are often based on knowledge gained from outcomes of decision trees used in previous experiences [13]. One Health research during emergency situation raises ethical dilemmas as there is an urgency to initiate the investigation and at the same time ensure appropriate approval is obtained from an ethics committee, particularly if the investigation occurs as part of a research project [14].

Researchers can create emergency response plans and consider the ethics component of the research without compromising research integrity [15,16]. While the general principles of research ethics used during normal conditions are similar, the unique circumstances of an emergency raise special considerations as the social fabric may be significantly disrupted and the functioning of Research Ethics Committees (RECs) may be compromised [17]. The RECs are charged with ensuring ethical oversight of research during disasters but may also face challenges, including difficulties maintaining standard operating procedures [18]. To date, limited knowledge exists regarding how RECs understand and apply research ethics principles as they review disaster research protocols, and whether and how they modify their procedures for disaster research review [18,19].

This project originated from the One Health Eastern Africa Research Training (OHEART) program which has the core mandate by ensuring that the purpose of building One Health research capacity through advanced training in Eastern African academic institutes located in Ethiopia, Kenya, and Tanzania [20]. This program was created within the Global One Health initiative (GOHi) at The Ohio State University (OSU), which aims to improve global health and implement the One Health approach in different parts of the world. To this end, under OHEART's training program, the One Health Ethics and Regulatory Procedures (OHEARP) project aims to identify ethical challenges and opportunities facing the timely approval and performance of One Health research in low- and middle-income countries (LMICs), with specific considerations for emergency situations.

This particular study was undertaken with the main aim of describing challenges faced by One Health researchers, REC members, and regulators during the review of One Health and emergency research proposals in African countries. Specific study aims included investigating the associations between demographic factors (e.g., participant professional role, country of One Health work, sex, age, and education) on the perception of ethical challenges faced during One Health research ethics review, and on the importance and feasibility of potential methods for improvement of ethics review procedures for One Health research during both non-emergency and emergency situations.

## 2 Material and methods

### 2.1 Study design, participant recruitment and data collection

For the current project, an anonymous online survey was conducted during March to June 2021 using Qualtrics software [21]. This study was deemed exempt from IRB review by The Ohio State University (OSU) Office of Responsible Research Practices (Study Number: 2021E0130), and received ethics approval from the Faculty of Veterinary Medicine Biosafety, Animal Use and Ethics Committee (FVM-BAUEC, Number: FVM BAUEC/2021/293) of the University of Nairobi (UoN), Kenyan National Commission for Science, Technology and Innovation (NACOSTI, License No: NACOSTI/P/21/9622), and National Animal Health Diagnostic & Investigation Center (NAHDIC, now Animal Health Institute/AHI) Animal Research Scientific and Ethics Review Committee (ARSERC/EC/018/11/03/2021). Prior to launching the survey, a series of revisions were made based on feedback from purposively selected GOHi researchers. These researchers included post-doctoral fellows, Ph.D. researchers and one of the project co-investigators. The survey was distributed to individuals whose email addresses were in the GOHi-OHEART email repository. A recruitment e-mail and participation flyer were sent to the individuals. To reach a wider scientific community, a Twitter account and website page were created for the OHEARP project and used to disseminate the survey as were the OHEARP research team's individual LinkedIn accounts and contacts. The survey was distributed to targeted organizations and institutions including all Kenyan RECs accredited by the National Commission for Science, Technology, and Innovation (NACOSTI), Kenya Veterinary Association and Kenya Medical Association, NAHDIC, Armauer Hansen Research Institute, Ethiopian Public Health Institute and the National Health Research Ethics Review Committee of Ethiopia. The survey was also shared with colleagues and relevant email listservs within various universities and industries in Africa, the United States, and Europe. Email reminders were sent after two weeks to those invited to participate. We utilized snowballing sampling for the study in an attempt to increase participation. As such, a formal sample size was not calculated given that we used multiple methods in our attempt to maximize the number of respondents.

For the purpose of the survey, One Health was defined according to the 2021 CDC definition as “a collaborative, multisectoral, and transdisciplinary approach working at the local, regional, national, and global levels to achieve optimal health outcomes recognizing the interconnection between people, animals, plants, and their shared environment” [22]. This definition was provided to participants several times during the survey. The survey contained a combination of open- and closed-ended questions divided into three main parts: demographics, challenges, and opportunities for improvement. The questionnaire included multiple-choice, and 5-point Likert scale questions, with possible responses ranging from 5 (extremely important) to 1 (not at all important). Time-to-completion was estimated to be between 15 and 45 min.

After a brief introduction to OHEARP, written consent to participate was requested and participants selected whether to consent to participate or not. Clicking the ‘do not consent’ button brought them immediately to an exit page. If they consented, they were directed to 15 closed-ended questions on their demographics. Next, participants were prompted to answer 1 of 3 sets of questions according to whether they were researchers, REC members, or regulators. The questions addressed the challenges participants encountered during research ethics review processes. Each role had a specific block of questions (18 questions for REC members and 12 each for researchers and regulators). Participants with multiple roles were asked to answer as many sets of questions as were relevant. Lastly, all participants were asked four questions regarding opportunities for and the feasibility of various strategies to improve interdisciplinary collaboration during ethics review processes for One Health research. The same questions were also asked regarding One Health research in emergency situations. Options provided to participants were gathered from the co-authors' experience, feedback received about the pilot survey as part of the validation process, factors previously reported in the literature [14,17,18,23,24], and expert opinion from colleagues. The full questionnaire is available as Supporting information. No identifying information was collected from the participants at any point in the survey.

### 2.2 Data analysis

For inclusion in analyses, respondents were required to be involved with One Health research in any African country in at least one role: either as a researcher, REC member, or regulator. Respondents were eligible to participate whether their current appointment was at an institution inside or outside Africa (i.e., they could be based in any country), but their One Health work had to be conducted in Africa. Surveys were exported from the Qualtrics software to Microsoft Excel for data management. Incomplete responses were excluded. Data were analyzed using descriptive statistics, including frequency and percentage for categorical variables, and mean and standard deviation (SD) for Likert scale-type variables.

Outcome (dependent) variables of interest from the study are listed on Table 1, and included the different challenges encountered during the review of One Health research (*n* = 8), and opportunities to improve the review of One Health research under non-emergency (*n* = 6) and emergency (*n* = 5) conditions. Our main predictor of interest was participants' professional role, which included REC Member, Regulator, One Health Researcher or Multiple Roles (defined as a participant having any two or all three of the included). For each of the dependent variables of interest, univariable models were used to investigate the separate effect of predictors of interest, including respondents' role, age, sex, experience in their selected role, level of education, and country (defined as the country where they were based, not necessarily the countries where their One Health research-related work or review occurred). As noted earlier, we had identified eight challenges encountered during ethics review processes (Table 1) and predictors for these were assessed by participants' role, multivariable linear mixed-effects models were built using a participant ID added as a random effect in all models to account for the lack of independence of responses among participants who had more than one role. For the improvement-related questions, since those were asked at the participant level and only once, multivariable linear models were built, with participants choosing more than one professional role considered “multiple roles” for the main predictor of interest. In the last case, participant responses were not repeated, and a random effect was not necessary.

**Table 1:** List of challenges and opportunities for review of One Health research and One Health research to be conducted specifically during emergency situations. These were provided in our survey instrument as options for ranking (by importance and by feasibility) by participants in the study.

List of challenges to timely and appropriate review of One Health proposals (*n* = 8)	List of strategies for opportunities to improve the review of One Health research (*n* = 6)	List of strategies for opportunities to improve the review of One Health research in emergency situations (*n* = 5)
• Insufficient time for proposal reviews	• Creation of an Interdisciplinary Committee	• Required training focused on emergencies
• Insufficient knowledge of One Health research	• Establishing a mandatory One Health review system by institutions	• Creation/use of Standard Operating Procedures for emergency proposals
• Insufficient protocols for emergency situations	• Creation/use of Standard Operating Procedures for One Health proposals	• Creation of an ad hoc ethics review committee for the emergency
• Inadequate guidelines/ protocols	• Required training for all ethics committee members/regulators	• Incentivizing reviewers specifically for emergency situations
• Insufficient knowledge of ethical issues specific to One Health research	• Improved channels for communication between researchers, reviewers, and regulators	• Improved channels for communication during emergencies between researchers and reviewers/regulators
• Lack of a multidisciplinary review board	• Incentivizing those who review One Health research	
• Large number of proposals		
• Lack of legal framework		

All data analysis was performed using R Statistical Software (https://www.R-project.org/, version 4.0.5), and *p* < 0.05 was considered as statistically significant, and 0.05 < *p* ≤ 0.1was considered a statistical trend. Given the main predictor of interest was professional role, models yielding those predictors as statistically significant are presented in the main manuscript body; other relevant models (in which other predictors were significant, or in which role was a statistical trend) are presented under Supporting information.

## 3 Results

### 3.1 Demographics

From a total of 214 participants who responded to the online survey, 170 participants (79.4%) met the eligibility criteria, and their demographics are presented in Table 2. These consisted of 68 One Health researchers, 18 REC members, 22 regulators, and 62 respondents reporting more than one role. Participants were engaged in One Health research in at least one of 13 African countries (Algeria, Cameroon, Côte d'Ivoire, Eritrea, Ethiopia, Guinea, Kenya, Liberia, Nigeria, Senegal, Somalia, South Africa, and Uganda), with most respondents reporting Ethiopia as their country of research focus (58.2%). Among the respondents, the majority were males (*n* = 70.6%). By age, 33.5% were 35–44 years old and 24.1% were aged 45–54 years. The majority of the respondents (65.9%) reported having a Doctorate degree, and 28.8% reported having a Master's degree as their highest degree (Table 2).

**Table 2:** Demographic characteristics for respondents who participated in the OHEARP survey.

Variable		Frequency *n* = 170	Percentage (%)
Sex	Male	120	70.6
	Female	50	29.4
Age	<35	39	23.0
	35–44	57	33.5
	45–54	41	24.1
	≥55	33	19.4
Highest education level	Bachelor's degree	9	5.3
	Master's degree	49	28.8
	Doctorate degree	112	65.9
Role	REC Member	18	10.6
	Regulator	22	12.9
	One Health Researcher	68	40.0
	Multiple Roles	62	36.5
Country where based	Ethiopia	99	58.2
	Kenya	32	18.8
	Other African Countries	22	13.0
	Non-African countries	17	10.0

### 3.2 Perception of One Health research ethics review challenges

Respondents' perceptions of the importance of specific challenges for the conduct, ethics review, and regulation of One Health research in Africa did not appear to vary according to examined demographic factors for seven of the eight challenges presented to participants. Only “Insufficient knowledge of One Health research” was perceived differently between males and females, with females perceiving it as less of a challenge (estimate: -0.42 [SE: 0.19]; p = 0.026; S1 Table) compared to males. “Insufficient knowledge of One Health research” was also perceived differently according to experience, with respondents reporting 10 or more years of experience perceiving it as a less important challenge compared to those with <6 years of experience in the job (−0.50 [0.23]; *p* = 0.0325; S1 Table).

### 3.3 Perceptions of the importance and feasibility of one health research ethics review improvement strategies

Participant role had a significant effect on the perception of the importance for “Establishing a mandatory One Health review system by institution.” Members of RECs perceived this strategy as less important compared to researchers (−0.73 [0.32]; *p* = 0.025; Table 3). Furthermore, respondents with a Master's degree as their highest level of education perceived the above mentioned strategy to be of more importance than those whose highest degree was a Bachelor's degree (1.23 [0.57]; *p* = 0.034; Table 3), while respondents based in non-African countries perceived it to be of lower importance compared to respondents based in Ethiopia (−1.23 [0.31]; *p* < 0.001; Table 3).

**Table 3:** Results from multivariable mixed effect regression model investigating the association between demographic variables and participants' perceived importance of “Establishing a mandatory One Health review system by institution” as a challenge for the review of One Health research. Statistically significant associations at the *p* < 0.05 level are marked with an asterisk (*).

Variable		Estimate (SE)	P-value
Role	One Health Researcher	Referent	
	REC Member	−0.73 (0.32)	0.025*
	Regulator	−0.06 (0.34)	0.85
	Multiple Roles	−0.31 (0.22)	0.17
Sex	Male	Referent	
	Female	−0.24 (0.21)	0.25
Age	<35	Referent	
	35–44	0.01 (0.28)	0.96
	45–54	−0.15 (0.30)	0.62
	≥55	0.08 (0.32)	0.81
Highest education level	Bachelor's Degree	Referent	
	Master's degree	1.23 (0.57)	0.03*
	Doctorate degree	0.58 (0.56)	0.30
Country where based	Ethiopia	Referent	
	Kenya	0.05 (0.26)	0.86
	Other African Countries	−0.25 (0.30)	0.40
	Non-African Countries	−1.23 (0.31)	0.000125*

Similarly, a country effect was observed for the importance of two improvement strategies, namely “Creation/use of SOPs for One Health proposals” and “Required training for all Committee members/Regulatory Body members” with lower importance perceived for non-African country-based participants compared to participants based in Ethiopia for both strategies (−0.79 [0.23]; p < 0.001 and − 0.94 [0.24]; *p* < 0.001, respectively; S2 and S3 Tables). Participants aged between 45 and 54 years also perceived “Required training” as of less importance (−0.54 [0.24]; *p* = 0.026; S3 Table) compared to participants <35 years old. Lastly, for the improvement “Incentivizing those who review One Health research”, respondents with multiple roles tended to rank this strategy as more important compared to researchers (0.42 [0.23]; *p* = 0.07; S4 Table), and respondents based in non-African countries tended to rank this as less important compared to Ethiopia-based respondents (−0.67 [0.32]; *p* = 0.04; S4 Table).

In regard to the feasibility of the six strategies presented in the survey for improvement of research ethics reviews, a tendency was found for REC members to perceive the strategy “Incentivizing those who review One health research” as more feasible when compared to researchers (0.59 [0.35]; *p* = 0.096; S5 Table). Furthermore, the feasibility of “Establishing a mandatory One Health review system by institutions” varied according to the country where respondents were based, with a decrease in perception of feasibility being observed for participants based in non-African countries (−0.92 [0.32]; *p* = 0.004; S6 Table) compared to respondents from Ethiopia. In addition, the feasibility perception for “Creation/use of SOPs for One Health proposals” depended on the respondent's education, with those whose highest degree was a Master's perceiving this strategy as more feasible when compared to those whose highest degree was a Bachelor's (1.15 [0.46]; *p* = 0.01; S7 Table). Finally, there was an effect of country for the strategy “Required training for Committee members/Regulatory Body members”, with participants based in non-African countries perceiving this strategy as less feasible compared to participants from Ethiopia (−0.90 [0.26]; *p* < 0.001; S8 Table).

### 3.4 Perceptions of the importance and feasibility of one health research ethics review improvement strategies during emergency situations

There were significant differences in perceptions of the importance of the strategy “Creation of an ad hoc ethics review committee for emergency situations” according to survey respondents' role and education level. Of note, respondents with multiple roles perceived this to be less important as an improvement strategy for emergency situations compared to researchers (−0.40 [0.31]; *p* = 0.02; Table 4). For this same strategy, individuals whose highest degree was a Master's (1.24 [0.51]; p = 0.02) and those with a Doctorate degree (1.09 [0.50]; *p* = 0.03) perceived the importance of this strategy to be more important compared to those whose highest degree was a Bachelor's (Table 4).

**Table 4:** Results from multivariable mixed effect regression model investigating the association between demographic variables and participants' perceived importance of “Creation of an ad hoc Committee” as an opportunity for improvement for the review of One Health research under emergency situations. Statistically significant associations at the *p* < 0.05 level are marked with an asterisk (*).

Variable		Estimate (SE)	P-value
Role	One Health Researcher	Referent	
	REC Member	3.19 (0.56)	0.0697
	Regulator	−0.51 (0.28)	0.19
	Multiple Roles	−0.40 (0.31)	0.0223*
Sex	Male	Reference	
	Female	0.07 (0.18)	0.67
Age	<35	Referent	
	35–44	−0.10 (0.25)	0.69
	45–54	−0.31 (0.27)	0.25
	≥55	0.31 (0.29)	0.29
Highest education level	Bachelor's Degree	Referent	
	Master's degree	1.24 (0.51)	0.0162*
	Doctorate degree	1.09 (0.50)	0.0306*
Country where based	Ethiopia	Referent	
	Kenya	−0.03 (0.23)	0.88
	Other African Countries	0.04 (0.26)	0.87
	Non-African Countries	−0.44 (0.27)	0.11

Participant role did not appear to be a significant predictor when examining the importance of other strategies for improvement of One Health research ethics reviews under emergency situations. Respondents based in non-African countries perceived “Creation/use of SOPs for One Health proposals” during emergencies to be of lower importance as an improvement strategy for the review process when compared to respondents from Ethiopia (−0.73 [0.26]; *p* = 0.006; S9 Table). Likewise, respondents based in non-African countries also perceived “Required training for all Committee members/regulators” to be of lower importance (−0.88 [0.25]; *p* < 0.001; S10 Table) compared to respondents from Ethiopia. The strategy “Incentivizing reviewers specifically for emergency situations” was perceived as a less important strategy for those between 45 and 55 years of age (−60 [0.30]; *p* = 0.04; S11 Table) compared to those aged <35 years and among those based in non-African countries (−0.98 [0.30]; *p* = 0.0015; S11 Table).

Regarding perceived feasibility of the five strategies presented, “Creation of an ad hoc Committee” was perceived as less feasible by REC members when compared to researchers (−0.66 [0.29]; *p* = 0.03; Table 5). In contrast, both those whose highest degree was a Master's and Doctorate degree holders perceived this option to be more feasible compared to those whose highest degree was a Bachelor's (1.31 [0.55]; *p* = 0.02 and 1.19 [0.55]; p = 0.03, respectively; Table 5). Finally, “Required training focused on emergency situations for all members” was the only other strategy with a significant predictor for feasibility, which was country of One Health work. Similarly to other strategies, respondents based in non-African countries ranked this strategy as less feasible when compared to Ethiopia-based participants (−0.89 [0.28]; *p* = 0.002; S12 Table).

**Table 5:** Results from multivariable mixed effect regression model investigating the association between demographic variables and participants' perceived feasibility of “Creation of ad hoc Committee” as an improvement opportunity for the review of One Health research under emergency situations. Statistically significant associations at the *p* < 0.05 level are marked with an asterisk (*).

Variable		Estimate (SE)	P-value
Role	One Health Researcher	Referent	
	REC Member	−0.66 (0.29)	0.0268*
	Regulator	−0.02 (0.36)	0.95
	Multiple Roles	−0.40 (0.20)	0.0533
Sex	Male	Referent	
	Female	0.16 (0.19)	0.39
Age	<35	Referent	
	35–44	−0.13 (0.26)	0.62
	45–54	−0.27 (0.28)	0.33
	≥55		0.47
Highest education level	Bachelor's degree	Referent	
	Master's degree	1.31 (0.55)	0.0199*
	Doctorate degree	1.19 (0.55)	0.0330*
Country where based	Ethiopia	Referent	
	Kenya	0.07 (0.24)	0.78
	Other African Countries	0.26 (0.28)	0.34
	Non-African Countries	−0.50 (0.29)	0.08

## 4 Discussion

Challenges to timely and appropriate review of One Health-related research had overall very high importance ranking scores in our study, with no role-specific differences found. A study conducted in one Eastern African country, Tanzania, and a published commentary by our research group came to similar conclusions as our survey, where lack of collaboration, inadequate turnaround time, and lack of communication within pertinent groups, were challenges to the timely ethics review of One Health research proposals [24,25]. In our study, participants from all three professional roles included had similar perceptions on all examined challenges to timely and appropriately ethics review of One Health research proposals, including insufficient time/knowledge of One Health or ethical issues specific to One Health, inadequate guidelines, large numbers of proposals, and lack of a multidisciplinary review board/legal framework/SOPs. The level of importance at which men and women, and participants with different levels of experience, ranked the challenge “insufficient knowledge of One Health research” varied in our study. In this case, women and experienced professionals (>10 years of experience in the role) perceived this challenge as a less important obstacle compared to men, and less experienced professionals. The reasons for these findings were not explored in the current study, but it could be hypothesized that professionals with more experience might have had more One Health-specific exposure and, therefore, may perceive this specific area of knowledge as less of an issue. In the case of female respondents perceiving “insufficient knowledge” as less of a challenge, this could be connected to current efforts to address the low representation of females in scientific research in most LMICs and the many trainings available to empower women as part of the United Nation's Sustainable Development Goals (SDGs) [[26], [27], [28]]. However, this hypothesis warrants further exploration.

Participants' professional role was an important predictor for perception of the importance placed on creating a mandatory review system for One Health research under non-emergency situations, with REC members perceiving this as less important compared to researchers. Likewise, the provision of incentives was perceived differently among stakeholders, with respondents with multiple roles considering this of higher importance than researchers, and the feasibility of incentives ranked higher by REC members than researchers. A possible explanation for this could be the nature of recognition and compensation in different professional appointments, which could also vary across institutions. Such factors were not examined in this survey. One could argue that these differences were expressed due to the fact that those involved with the process from multiple angles would appreciate and potentially be motivated by incentives. Other common demographic factors affecting the importance of improvement factors appeared to be education and country where respondents were based. Those whose highest degree was a Master's assigned higher importance to the strategies of creating mandatory review systems as well as developing SOPs compared to those whose highest degree was a Bachelor's. This could be because training for higher degrees may have exposed learners to materials and discussions that exemplified the complexities of these review sessions, and thereby led to awareness of the need for structured guidelines for successful outcomes. Country of work also appeared important, with respondents based in non-African countries generally ranking several improvement strategies, including the creation of mandatory review systems, creation of SOPs, and mandatory training, lower in level of importance and feasibility compared to respondents from African countries. These comparisons were significantly different for Ethiopia-based participants, who overall appeared to significantly and consistently rank more highly the importance and feasibility of improvement strategies. This finding should be interpreted with caution since it might be a reflection of the on-going collaborative efforts through the OHEART program and GOHi mentioned in the Introduction which have been implemented primarily in Ethiopia. This could make this specific population more likely to be positive about the importance and feasibility of improvement given the success of similar efforts in these programs.

Respondents also differed in their perceptions about the importance of the creation of ad hoc committees during emergencies, with respondents occupying multiple roles attributing a lower importance compared to researchers, and those whose highest degree was at least a Master's degree perceiving ad hoc committees to be of higher importance compared to those whose highest degree was a Bachelor's. This could be explained by the fact that researchers feel strongly about issues with delays in research ethics approvals in humanitarian/ emergency situations [29], while those with multiple roles could potentially perceive that, in emergency situations, other activities would require prioritization compared to the speed of research itself. Similar findings were found with the feasibility assessment questions, where the creation of ad hoc committees was ranked as less feasible by REC members compared to researchers. This result may be explained by a perceived lack of capacity and resources to realize this as a less feasible strategy. During emergency situations, strategies including mandatory training, creation of SOPs and provision of incentives were also perceived as less important by respondents based in non-African countries compared to respondents from Ethiopia; which also could be explained by positive attitudes from this specific population and successful experiences by individuals involved in One Health programs through current efforts [12,30]. This finding could also be explained by a potential lack of recognition of local opportunities from the perspectives of those based in non-African countries (e.g. a “Westernized perspective”), or due to past experiences that were not successful [31].

Although specifics on the type of strategy best suited to support timely One Health research reviews during emergency situations varied with participants' demographics, all proposed strategies were ranked overall high in importance (Supporting Information S13 and S14 Tables), which was not surprising. A similar study conducted in Uganda outlined the necessity of appropriate preparedness strategies, including implementation and strengthening of RECs in LMICs, and education and training in research ethics [32]. Another study reviewing current One Health research practices supported our study's results by proposing similar strategies for a fully comprehensive and trained ethics committee [33]. Their strategies include expanding educational capacity starting at the university level and incorporating multidisciplinary ethics review processes to create an all-encompassing up-to-date operating procedure. Our study and those cited here were in agreement over the need to improve guidance and training to enhance review quality and response time of One Health research proposals. However, prioritizing strategies to initiate improvement processes for One Health research projects is not a simple task. An evaluation of ethics review processes in developing countries found that LMICs follow a Westernized approach to ethics review of research, another challenge not explicitly discussed in the current survey [31]. Potential improvements proposed by others include updating policies and training surrounding research and emergency scenarios to better reflect the current socio-economic and cultural environment [34]. To further this conclusion, a study reviewed capacity building for research ethics in Sub-Saharan Africa specifically for NIH Fogarty-funded projects and found that research ethics training was lacking, inconsistent across the projects and did not reflect the best current practices of research ethics [35]. A recent evaluation of research ethics systems in Latin America and the Caribbean found similar challenges and limitations [36]. Our survey connects the described studies by similarly concluding that updating SOPs and training materials for RECs, specifically to include One Health-related research, could be helpful strategies in revising current practices; but these strategies might face resistance from stakeholders. The existing research illustrates that systemic changes are needed to ethics review processes in LMIC, and our survey results supplement and support this demand by summarizing potential pathways to accomplish development in this area. In response to an emergency, ethics review may need to be carried out more rapidly, but the review should maintain high quality and rigorous evaluation as appropriate to the nature of the research. Evidence from ethics review during the COVID-19 pandemic, at least in the U.S., has shown that ethics committees can adapt their procedures to take account of the need to rapidly review an influx of emergency-related research [37].

## 5 Future research

Since One Health is an evolving field, navigating socio-ethical public health considerations will require additional research that goes beyond the current enterprise. Our survey results show the need for changes to optimize how One Health-related projects are currently reviewed. However, the feasibility of the strategies listed and steps for implementation need to be further investigated in order to improve the review and execution of One Health research, especially during emergency scenarios. Some steps that can be taken include drafting policies at the local, national, and global levels that clearly outline the training and personnel required to conduct ethics reviews and provide SOPs for research during emergencies. Another potential area of future research would be empirical investigations to better understand social and political challenges with One Health research ethics review in Africa, similar to a conceptual and documentation study conducted in Australia [34]. Creating a more streamlined One Health research ethics infrastructure, including policies, SOPs, and review member qualifications could be a solution for identified barriers outlined in our study and in accordance with existing research.

Additional potential areas for future research include replicating our survey with an expanded pool of respondents as we had only four respondents from French-speaking African countries. The experience, procedures, and opinions of individuals from different countries and cultures, such as Francophone or Asian countries, could differ from those given by the respondents to our survey. To address this, the survey would need to be translated into other languages and disseminated more widely through additional networks. Research into the views of participant communities where One Health research is conducted could also provide important insight into how ethical issues are and should be addressed [38]. Lastly, qualitative interviews would provide further information on the challenges and implementation of different strategies to address them. Our team is currently conducting key informant interviews to elaborate on our findings and to explore in greater depth the ethical challenges found in our survey.

## 6 Limitations

The study had limitations that are common with online survey-based studies. First, in order to keep the survey within a reasonable duration, we provided specific challenges for respondents to rank, which may not have included all relevant ones. We attempted to address this by allowing respondents to write in additional challenges which will be published elsewhere. We acknowledge that lack of time could be an issue when seeking additional details with open-ended questions. Third, some questions may have been written in ways that could have led to information bias.

Respondents for our study were recruited electronically, which has limitations. This approach allowed us to recruit a robust number of participants for a relatively low cost and to reach respondents with ease and modest geographical coverage. However, the online nature of the survey precluded calculating a response rate as it is impossible to tell how many respondents received the survey. As such, we were unable to understand the representativeness of our respondents as it relates to One Health research stakeholders in Africa. Most respondents resided in African countries with limited internet access and financial challenges which may have deterred some people from completing the survey and biased its conclusions. On the other hand, we also acknowledge that researchers based outside of Africa but with their One Health research still focused on Africa may have a different experience with ethics processes. A sub-analysis may be needed to elucidate the specific challenges they face. The project advertised the survey in various ways in an effort to minimize such bias [39,40]. Lastly, another limitation noted was the small representation from western and North African countries, and specifically from French-speaking African countries; this limits the generalizability of our results across Africa. We were also unable to separately analyze responses from researchers primarily residing in African countries versus others (e.g., Europe/America), which could be interesting to pursue in the future.

## 7 Conclusions

Our study contributes to the small number of available studies discussing the ethical review of One Health research. This study included stakeholders with different roles in the ethics review of One Health research in an African context, exploring both the challenges experienced with review and compiling the perceptions of One Health researchers, REC members and regulators on potential changes that could improve One Health research ethics. Efficient and timely One Health research ethics review can be impacted by multifaceted issues such as lack of time for reviewing research protocols, insufficient knowledge of One Health research and its associated ethical issues, and lack of a multidisciplinary review board. Our study results emphasize the need to implement and evaluate strategies to improve the ethics review of One Health and emergency research in Africa.

## Funding

This work was supported by the 10.13039/100000002National Institutes of Health-10.13039/100000061Fogarty International Center (NIH-FIC), [grant number: 3D43TW008650-08S1].

## CRediT authors' contribution statement

**Kuastros Belaynehe**: Conceptualization, data curation, designing-modifying & distributing the questionnaire, data analysis & interpretation, writing-original draft, writing - review & editing. **Joseph Nguta**: designing-modifying & distributing the questionnaire, writing - review & editing. **Melanie Lopez**: data curation, designing-modifying & distributing the questionnaire, writing - review & editing. **Jinjian Mu**: data curation, data analysis & interpretation, writing - review & editing. **Dónal O'Mathúna**: Conceptualization, funding acquisition, designing-modifying & distributing the questionnaire, writing - review & editing. **Getnet Yimer**: Conceptualization, funding acquisition, designing-modifying & distributing the questionnaire, writing - review & editing. **Andréia Arruda**: Conceptualization, data curation, funding acquisition, designing-modifying & distributing the questionnaire, data analysis & interpretation, writing - review & editing.

## Declaration of competing interest

The authors declare that the study was conducted in the absence of any commercial or financial relationships that could be construed as a potential conflict of interest.

## Data Availability

All summary data generated during this work are included in this published article. Original data is available under Supporting information files.

## Appendix A Supplementary data

Supplementary material 1: Results from multivariable mixed effect regression model investigating the association between demographic variables and participant’s perceived **importance** of “Insufficient knowledge of One Health research” as a **challenge** for the review of One Health research. Statistically significant associations at the p<0.05 level are marked with an asterisk (*). Image 1

Supplementary material 2: Results from multivariable mixed effect regression model investigating the association between demographic variables and participants’ perceived **importance** of “Creation/use of SOPs for One Health proposals” as an **improvement** opportunity for the review of One Health research under **non-emergency situations**. Statistically significant associations at the p<0.05 level are marked with an asterisk (*). Image 2

Supplementary material 3: Results from multivariable mixed effect regression model investigating the association between demographic variables and participants’ perceived **importance** of “Required training for all Committee members/regulatory Body members” as an **improvement** opportunity for the review of One Health research under **non-emergency situations**. Statistically significant associations at the p<0.05 level are marked with an asterisk (*). Image 3

Supplementary material 4: Results from multivariable mixed effect regression model investigating the association between demographic variables and participants’ perceived **importance** of “Incentivizing those who review One Health research” as an **improvement** opportunity for the review of One Health research under **non-emergency situations.** Statistically significant associations at the p<0.05 level are marked with an asterisk (*). Image 4

Supplementary material 5: Results from multivariable mixed effect regression model investigating the association between demographic variables and participants’ perceived **feasibility** of “Incentivizing those who review One Health research” as an **improvement** opportunity for the review of One Health research under **non-emergency situations**. Statistically significant associations at the p<0.05 level are marked with an asterisk (*). Image 5

Supplementary material 6: Results from multivariable mixed effect regression model investigating the association between demographic variables and participants’ perceived **feasibility** of “Establishing a mandatory One Health review system by institutions” as an **improvement** opportunity for the review of One Health research under **non-emergency situations**. Statistically significant associations at the p<0.05 level are marked with an asterisk (*). Image 6

Supplementary material 7: Results from multivariable mixed effect regression model investigating the association between demographic variables and participants’ perceived **feasibility** of “Creation/use of SOPs for One Health proposals” as an **improvement** opportunity for the review of One Health research under **non-emergency situations**. Statistically significant associations at the p<0.05 level are marked with an asterisk (*). Image 7

Supplementary material 8: Results from multivariable mixed effect regression model investigating the association between demographic variables and participants’ perceived **feasibility** of “Required training for all Committee members/Regulatory Body members” as an **improvement** opportunity for the review of One Health research under **non-emergency situations**. Statistically significant associations at the p<0.05 level are marked with an asterisk (*). Image 8

Supplementary material 9: Results from multivariable mixed effect regression model investigating the association between demographic variables and participants’ perceived **importance** of “Creation/use of SOPs for One Health proposals” as an **improvement** opportunity for the review of One Health research under **emergency situations**. Statistically significant associations at the p<0.05 level are marked with an asterisk (*). Image 9

Supplementary material 10: Results from multivariable mixed effect regression model investigating the association between demographic variables and participants’ perceived **importance** of “Required training focused on emergency situations for all members” as an **improvement** opportunity for the review of One Health research under **emergency situations**. Statistically significant associations at the p<0.05 level are marked with an asterisk (*). Image 10

Supplementary material 11: Results from multivariable mixed effect regression model investigating the association between demographic variables and participants’ perceived **importance** of  “Incentivizing reviewers” as an **improvement** opportunity for the review of One Health research under **emergency situations**. Statistically significant associations at the p<0.05 level are marked with an asterisk (*). Image 11

Supplementary material 12: Results from multivariable mixed effect regression model investigating the association between demographic variables and participants’ perceived **feasibility** of “Required training focused on emergency situations for all members” as an **improvement** opportunity for the review of One Health research under **emergency situations**. Statistically significant associations at the p<0.05 level are marked with an asterisk (*). Image 12

Supplementary material 13: Strategies to **improve** reviews of One Health research by Researchers, Research Ethics Committee Members, and Regulators. Scores are represented in a Likert scale from 1-5 (5 representing highest agreement); and means (SD) are presented for the “Professional Role” columns. P-values are obtained from multivariable mixed effect models (which include other demographic variables, namely age, education, country of origin, sex and experience) and represent statistical significance for at least one role. Image 13

Supplementary material 14: Strategies to **improve** reviews of One Health research by Researchers, Research Ethics Committee Members, and Regulators under **emergency situations** (e.g. disasters, pandemics, etc.). Scores are represented in a Likert scale from 1-5; (5 representing highest agreement) and means (SD) are presented for the “Professional Role” columns. P-values are obtained from multivariable mixed effect models (which include other demographic variables, namely age, education, country of origin, sex and experience) and represent statistical significance for at least one role. Image 14

Supplementary material 15: OHEARP_Survey Image 15
