# Supplementary material for: One Health research ethics review processes in African countries: Challenges and opportunities
Source: One Health. 2024 Mar 22;18:100716. doi: 10.1016/j.onehlt.2024.100716 (PMC11247289; doi:10.1016/j.onehlt.2024.100716)
Supplement: Supplementary file 1 — Supplementary material 1: Results from multivariable mixed effect regression model investigating the association between demographic variables and participant’s perceived importance of “Insufficient knowledge of One Health research” as a challenge for the review of One Health research. Statistically significant associations at the p<0.05 level are marked with an asterisk (*). [file mmc1.docx]

**S1 Table.** Results from multivariable mixed effect regression model investigating the association between demographic variables and participant’s perceived **importance** of “Insufficient knowledge of One Health research” as a **challenge** for the review of One Health research. Statistically significant associations at the p<0.05 level are marked with an asterisk (*).

| Variable | | Estimate (SE) | P-value |
| --- | --- | --- | --- |
| Role | |  |  |
|  | One Health Researcher | Referent |  |
|  | REC Member | -0.08 (0.19) | 0.66 |
|  | Regulator | 0.34 (0.20) | 0.08 |
| Sex | |  |  |
|  | Male | Referent |  |
|  | Female | -0.42 (0.19) | 0.0262* |
| Age | |  |  |
|  | <35 | Referent |  |
|  | 35-44 | -0.34 (0.24) | 0.16 |
|  | 45-54 | 0.17 (0.26) | 0.52 |
|  | ≥55 | 0.04 (0.30) | 0.88 |
| Highest education level | |  |  |
|  | Bachelor’s Degree | Referent |  |
|  | Master’s degree | 0.14 (0.46) | 0.75 |
|  | Doctorate degree | -0.22 (0.45) | 0.63 |
| Country of work | |  |  |
|  | Ethiopia | Referent |  |
|  | Kenya | 0.31 (0.24) | 0.19 |
|  | Other African Countries | 0.10 (0.24) | 0.67 |
|  | Not African Countries | 0.23 (0.28) | 0.41 |
| Experience | |  |  |
|  | < 6 years | Referent |  |
|  | 6-10 years | 0.25 (0.18) | 0.19 |
|  | Over 10 years | -0.50 (0.23) | 0.0325* |
