# Supplementary material for: One Health research ethics review processes in African countries: Challenges and opportunities
Source: One Health. 2024 Mar 22;18:100716. doi: 10.1016/j.onehlt.2024.100716 (PMC11247289; doi:10.1016/j.onehlt.2024.100716)
Supplement: Supplementary file 2 — Supplementary material 2: Results from multivariable mixed effect regression model investigating the association between demographic variables and participants’ perceived importance of “Creation/use of SOPs for One Health proposals” as an improvement opportunity for the review of One Health research under non-emergency situations. Statistically significant associations at the p<0.05 level are marked with an asterisk (*). [file mmc2.docx]

**S2 Table.** Results from multivariable mixed effect regression model investigating the association between demographic variables and participants’ perceived **importance** of “Creation/use of SOPs for One Health proposals” as an **improvement** opportunity for the review of One Health research under **non-emergency situations**. Statistically significant associations at the p<0.05 level are marked with an asterisk (*).

| Variable | | Estimate (SE) | P-value |
| --- | --- | --- | --- |
| Role | |  |  |
|  | One Health Researcher | Referent |  |
|  | REC Member | -0.09 (0.24) | 0.70 |
|  | Regulator | -0.22 (0.25) | 0.39 |
|  | Multiple Roles | 0.01 (0.16) | 0.93 |
| Age | |  |  |
|  | <35 | Referent |  |
|  | 35-44 | -0.20 (0.21) | 0.34 |
|  | 45-54 | 0.15 (0.22) | 0.48 |
|  | ≥55 | 0.09 (0.23) | 0.70 |
| Sex | |  |  |
|  | Male | Referent |  |
|  | Female | 0.10 (0.15) | 0.52 |
| Highest education level | |  |  |
|  | Bachelor’s Degree | Referent |  |
|  | Master’s degree | -0.13 (0.42) | 0.76 |
|  | Doctorate degree | -0.50 (0.41) | 0.23 |
| Country of work | |  |  |
|  | Ethiopia | Referent |  |
|  | Kenya | -0.23 (0.19) | 0.23 |
|  | Other African Countries | -0.15 (0.22) | 0.51 |
|  | Not African Countries | -0.79 (0.23) | 0.000689* |
