# Supplementary material for: One Health research ethics review processes in African countries: Challenges and opportunities
Source: One Health. 2024 Mar 22;18:100716. doi: 10.1016/j.onehlt.2024.100716 (PMC11247289; doi:10.1016/j.onehlt.2024.100716)
Supplement: Supplementary file 3 — Supplementary material 3: Results from multivariable mixed effect regression model investigating the association between demographic variables and participants’ perceived importance of “Required training for all Committee members/regulatory Body members” as an improvement opportunity for the review of One Health research under non-emergency situations. Statistically significant associations at the p<0.05 level are marked with an asterisk (*). [file mmc3.docx]

**S3 Table.** Results from multivariable mixed effect regression model investigating the association between demographic variables and participants’ perceived **importance** of “Required training for all Committee members/regulatory Body members” as an **improvement** opportunity for the review of One Health research under **non-emergency situations**. Statistically significant associations at the p<0.05 level are marked with an asterisk (*).

| Variable | | Estimate (SE) | P-value |
| --- | --- | --- | --- |
| Role | |  |  |
|  | One Health Researcher | Referent |  |
|  | REC Member | -0.13 (0.26) | 0.61 |
|  | Regulator | 0.003 (0.27) | 0.99 |
|  | Multiple Roles | -0.05 (0.18) | 0.77 |
| Age | |  |  |
|  | <35 | Referent |  |
|  | 35-44 | -0.33 (0.22) | 0.14 |
|  | 45-54 | -0.54 (0.24) | 0.026* |
|  | ≥55 | -0.37 (0.25) | 0.15 |
| Sex | |  |  |
|  | Male | Referent |  |
|  | Female | 0.01 (0.16) | 0.93 |
| Highest education level | |  |  |
|  | Bachelor’s Degree | Referent |  |
|  | Master’s degree | 0.03 (0.45) | 0.94 |
|  | Doctorate degree | -0.06 (0.44) | 0.88 |
| Country of work | |  |  |
|  | Ethiopia | Referent |  |
|  | Kenya | -0.30 (0.21) | 0.15 |
|  | Other African Countries | -0.10 (0.24) | 0.66 |
|  | Not African Countries | -0.94 (0.24) | 0.000208* |
