# Supplementary material for: One Health research ethics review processes in African countries: Challenges and opportunities
Source: One Health. 2024 Mar 22;18:100716. doi: 10.1016/j.onehlt.2024.100716 (PMC11247289; doi:10.1016/j.onehlt.2024.100716)
Supplement: Supplementary file 6 — Supplementary material 6: Results from multivariable mixed effect regression model investigating the association between demographic variables and participants’ perceived feasibility of “Establishing a mandatory One Health review system by institutions” as an improvement opportunity for the review of One Health research under non-emergency situations. Statistically significant associations at the p<0.05 level are marked with an asterisk (*). [file mmc6.docx]

**S6 Table.** Results from multivariable mixed effect regression model investigating the association between demographic variables and participants’ perceived **feasibility** of “Establishing a mandatory One Health review system by institutions” as an **improvement** opportunity for the review of One Health research under **non-emergency situations**. Statistically significant associations at the p<0.05 level are marked with an asterisk (*).

| Variable | | Estimate (SE) | P-value |
| --- | --- | --- | --- |
| Role | |  |  |
|  | One Health Researcher | Referent |  |
|  | REC Member | -0.48 (0.33) | 0.15 |
|  | Regulator | -0.17 (0.35) | 0.62 |
|  | Multiple Roles | -0.35 (0.23) | 0.13 |
| Age | |  |  |
|  | <35 | Referent |  |
|  | 35-44 | 0.09 (0.29) | 0.74 |
|  | 45-54 | -0.09 (0.31) | 0.76 |
|  | ≥55 | 0.29 (0.33) | 0.38 |
| Sex | |  |  |
|  | Male | Referent |  |
|  | Female | -0.15 (0.21) | 0.49 |
| Highest education level | |  |  |
|  | Bachelor’s Degree | Referent |  |
|  | Master’s degree | 0.68 (0.58) | 0.24 |
|  | Doctorate degree | 0.24 (0.57) | 0.67 |
| Country of work | |  |  |
|  | Ethiopia | Referent |  |
|  | Kenya | 0.28 (0.27) | 0.30 |
|  | Other African Countries | 0.31 (0.32) | 0.33 |
|  | Not African Countries | -0.92 (0.32) | 0.00423* |
