# Supplementary material for: One Health research ethics review processes in African countries: Challenges and opportunities
Source: One Health. 2024 Mar 22;18:100716. doi: 10.1016/j.onehlt.2024.100716 (PMC11247289; doi:10.1016/j.onehlt.2024.100716)
Supplement: Supplementary file 7 — Supplementary material 7: Results from multivariable mixed effect regression model investigating the association between demographic variables and participants’ perceived feasibility of “Creation/use of SOPs for One Health proposals” as an improvement opportunity for the review of One Health research under non-emergency situations. Statistically significant associations at the p<0.05 level are marked with an asterisk (*). [file mmc7.docx]

**S7 Table.** Results from multivariable mixed effect regression model investigating the association between demographic variables and participants’ perceived **feasibility** of “Creation/use of SOPs for One Health proposals” as an **improvement** opportunity for the review of One Health research under **non-emergency situations**. Statistically significant associations at the p<0.05 level are marked with an asterisk (*).

| Variable | | Estimate (SE) | P-value |
| --- | --- | --- | --- |
| Role | |  |  |
|  | One Health Researcher | Referent |  |
|  | REC Member | 0.13 (0.26) | 0.60 |
|  | Regulator | 0.30 (0.28) | 0.28 |
|  | Multiple Roles | 0.14 (0.18) | 0.42 |
| Age | |  |  |
|  | <35 | Referent |  |
|  | 35-44 | -0.06 (0.23) | 0.79 |
|  | 45-54 | -0.08 (0.24) | 0.75 |
|  | ≥55 | 0.07 (0.26) | 0.79 |
| Sex | |  |  |
|  | Male | Referent |  |
|  | Female | -0.001 (0.16) | 0.99 |
| Highest education level | |  |  |
|  | Bachelor’s Degree | Referent |  |
|  | Master’s degree | 1.15 (0.46) | 0.0132* |
|  | Doctorate degree | 0.75 (0.44) | 0.09 |
| Country of work | |  |  |
|  | Ethiopia | Referent |  |
|  | Kenya | -0.18 (0.21) | 0.41 |
|  | Other African Countries | 0.23 (0.23) | 0.32 |
|  | Not African Countries | -0.48 (0.25) | 0.054 |
