# Supplementary material for: One Health research ethics review processes in African countries: Challenges and opportunities
Source: One Health. 2024 Mar 22;18:100716. doi: 10.1016/j.onehlt.2024.100716 (PMC11247289; doi:10.1016/j.onehlt.2024.100716)
Supplement: Supplementary file 8 — Supplementary material 8: Results from multivariable mixed effect regression model investigating the association between demographic variables and participants’ perceived feasibility of “Required training for all Committee members/Regulatory Body members” as an improvement opportunity for the review of One Health research under non-emergency situations. Statistically significant associations at the p<0.05 level are marked with an asterisk (*). [file mmc8.docx]

**S8 Table.** Results from multivariable mixed effect regression model investigating the association between demographic variables and participants’ perceived **feasibility** of “Required training for all Committee members/Regulatory Body members” as an **improvement** opportunity for the review of One Health research under **non-emergency situations**. Statistically significant associations at the p<0.05 level are marked with an asterisk (*).

| Variable | | Estimate (SE) | P-value |
| --- | --- | --- | --- |
| Role | |  |  |
|  | One Health Researcher | Referent |  |
|  | REC Member | -0.05 (0.27) | 0.84 |
|  | Regulator | -0.28 (0.29) | 0.34 |
|  | Multiple Roles | -0.14 (0.19) | 0.46 |
| Age | |  |  |
|  | <35 | Referent |  |
|  | 35-44 | -0.27 (0.24) | 0.25 |
|  | 45-54 | -0.50 (0.25) | 0.053 |
|  | ≥55 | -0.04 (0.27) | 0.88 |
| Sex | |  |  |
|  | Male | Referent |  |
|  | Female | -0.31 (0.17) | 0.08 |
| Highest education level | |  |  |
|  | Bachelor’s Degree | Referent |  |
|  | Master’s degree | 0.53 (0.48) | 0.27 |
|  | Doctorate degree | 0.44 (0.46) | 0.34 |
| Country of work | |  |  |
|  | Ethiopia | Referent |  |
|  | Kenya | -0.32 (0.22) | 0.15 |
|  | Other African Countries | -0.08 (0.25) | 0.76 |
|  | Not African Countries | -0.90 (0.26) | 0.000747* |
