# Supplementary material for: One Health research ethics review processes in African countries: Challenges and opportunities
Source: One Health. 2024 Mar 22;18:100716. doi: 10.1016/j.onehlt.2024.100716 (PMC11247289; doi:10.1016/j.onehlt.2024.100716)
Supplement: Supplementary file 9 — Supplementary material 9: Results from multivariable mixed effect regression model investigating the association between demographic variables and participants’ perceived importance of “Creation/use of SOPs for One Health proposals” as an improvement opportunity for the review of One Health research under emergency situations. Statistically significant associations at the p<0.05 level are marked with an asterisk (*). [file mmc9.docx]

**S9 Table.** Results from multivariable mixed effect regression model investigating the association between demographic variables and participants’ perceived **importance** of “Creation/use of SOPs for One Health proposals” as an **improvement** opportunity for the review of One Health research under **emergency situations**. Statistically significant associations at the p<0.05 level are marked with an asterisk (*).

| Variable | | Estimate (SE) | P-value |
| --- | --- | --- | --- |
| Role | |  |  |
|  | One Health Researcher | Referent |  |
|  | REC Member | -0.14 (0.28) | 0.61 |
|  | Regulator | 0.11 (0.31) | 0.72 |
|  | Multiple Roles | -0.06 (0.19) | 0.73 |
| Age | |  |  |
|  | <35 | Referent |  |
|  | 35-44 | 0.10 (0.25) | 0.68 |
|  | 45-54 | 0.16 (0.26) | 0.54 |
|  | ≥55 | 0.26 (0.28) | 0.36 |
| Sex | |  |  |
|  | Male | Referent |  |
|  | Female | 0.28 (0.18) | 0.12 |
| Highest education level | |  |  |
|  | Bachelor’s Degree | Referent |  |
|  | Master’s degree | 0.27 (0.50) | 0.58 |
|  | Doctorate degree | -0.08 (0.49) | 0.87 |
| Country of work | |  |  |
|  | Ethiopia | Referent |  |
|  | Kenya | -0.09 (0.23) | 0.69 |
|  | Other African Countries | 0.07 (0.26) | 0.79 |
|  | Not African Countries | -0.73 (0.26) | 0.0061* |
