# Supplementary material for: One Health research ethics review processes in African countries: Challenges and opportunities
Source: One Health. 2024 Mar 22;18:100716. doi: 10.1016/j.onehlt.2024.100716 (PMC11247289; doi:10.1016/j.onehlt.2024.100716)
Supplement: Supplementary file 11 — Supplementary material 11: Results from multivariable mixed effect regression model investigating the association between demographic variables and participants’ perceived importance of “Incentivizing reviewers” as an improvement opportunity for the review of One Health research under emergency situations. Statistically significant associations at the p<0.05 level are marked with an asterisk (*). [file mmc11.docx]

**S11 Table.** Results from multivariable mixed effect regression model investigating the association between demographic variables and participants’ perceived **importance** of “Incentivizing reviewers” as an **improvement** opportunity for the review of One Health research under **emergency situations**. Statistically significant associations at the p<0.05 level are marked with an asterisk (*).

| Variable | | Estimate (SE) | P-value |
| --- | --- | --- | --- |
| Role | |  |  |
|  | One Health Researcher | Referent |  |
|  | REC Member | 0.30 (0.32) | 0.36 |
|  | Regulator | -0.14 (0.34) | 0.68 |
|  | Multiple Roles | 0.08 (0.22) | 0.72 |
| Age | |  |  |
|  | <35 | Referent |  |
|  | 35-44 | -0.14 (0.28) | 0.62 |
|  | 45-54 | -0.60 (0.30) | 0.04577* |
|  | ≥55 | -0.08 (0.32) | 0.81 |
| Sex | |  |  |
|  | Male | Referent |  |
|  | Female | -0.22 (0.20) | 0.28 |
| Highest education level | |  |  |
|  | Bachelor’s Degree | Referent |  |
|  | Master’s degree | 0.73 (0.56) | 0.20 |
|  | Doctorate degree | 0.04 (0.55) | 0.93 |
| Country of work | |  |  |
|  | Ethiopia | Referent |  |
|  | Kenya | 0.19 (0.26) | 0.48 |
|  | Other African Countries | -0.08 (0.30) | 0.80 |
|  | Not African Countries | -0.98 (0.30) | 0.00148* |
