# Supplementary material for: One Health research ethics review processes in African countries: Challenges and opportunities
Source: One Health. 2024 Mar 22;18:100716. doi: 10.1016/j.onehlt.2024.100716 (PMC11247289; doi:10.1016/j.onehlt.2024.100716)
Supplement: Supplementary file 13 — Supplementary material 13: Strategies to improve reviews of One Health research by Researchers, Research Ethics Committee Members, and Regulators. Scores are represented in a Likert scale from 1-5 (5 representing highest agreement); and means (SD) are presented for the “Professional Role” columns. P-values are obtained from multivariable mixed effect models (which include other demographic variables, namely age, education, country of origin, sex and experience) and represent statistical significance for at least one role. [file mmc13.docx]

**S13 Table.** Strategies to **improve** reviews of One Health research by Researchers, Research Ethics Committee Members, and Regulators. Scores are represented in a Likert scale from 1-5 (5 representing highest agreement); and means (SD) are presented for the “Professional Role” columns. P-values are obtained from multivariable mixed effect models (which include other demographic variables, namely age, education, country of origin, sex and experience) and represent statistical significance for at least one role.

|  |  | | | | **95% Confidence Interval** | | | | | | **P-value** |
| --- | --- | --- | --- | --- | --- | --- | --- | --- | --- | --- | --- |
|  | **Professional Role** | | | | **One Health Researcher vs REC Member** | **One Health Researcher vs Regulator** | **One Health Researcher vs Multiple Roles** | **REC Member vs Regulator** | **REC Member vs Multiple Roles** | **Regulator vs Multiple Roles** |  |
|  | **One Health Researcher** | **REC Member** | **Regulator** | **Multiple Roles** |  |  |  |  |  |  |  |
| **Importance** |  |  |  |  |  |  |  |  |  |  |  |
| Creation of an Interdisciplinary Committee | 4.27 (0.72) | 3.93 (0.70) | 4.12 (1.11) | 4.11 (0.96) | (-0.36, 1.01) | (-0.66, 0.83) | (-0.27, 0.68) | (-1.13, 0.66) | (-0.80, 0.56) | (-0.61, 0.85) | 0.528 |
| Establishing a mandatory One Health review system by institutions | 3.66 (1.21) | 3.21 (0.97) | 3.71 (1.10) | 3.48 (1.09) | (-0.11, 1.58) | (-0.84, 0.96) | (-0.28, 0.89) | (-1.76, 0.42) | (-1.27, 0.41) | (-0.63, 1.12) | 0.435 |
| Creation/use of Standard Operating Procedures (SOPs) for One Health proposals | 4.12 (0.82) | 4.14 (0.66) | 4.24 (0.83) | 4.29 (0.76) | (-0.53, 0.72) | (-0.45, 0.88) | (-0.44, 0.41) | (-0.68, 0.93) | (-0.73, 0.51) | (-0.88, 0.41) | 0.722 |
| Required training for all Committee members/Regulatory Body members | 4.06 (0.98) | 4.07 (0.47) | 4.41 (0.80) | 4.16 (0.82) | (-0.54, 0.80) | (-0.72, 0.71) | (-0.41, 0.52) | (-1.00, 0.73) | (-0.75, 0.59) | (-0.64, 0.75) | 0.467 |
| Improved channels for communication between researchers and reviewers/Regulatory Body members | 4.12 (0.69) | 4.00 (0.68) | 4.29 (0.92) | 3.98 (0.72) | (-0.43, 0.77) | (-0.76, 0.52) | (-0.31, 0.52) | (-1.07, 0.49) | (-0.67, 0.54) | (-0.40, 0.85) | 0.439 |
| Incentivizing those who review One Health research | 3.29 (1.14) | 3.50 (1.22) | 3.65 (1.22) | 3.69 (1.02) | (-0.98, 0.77) | (-1.25, 0.62) | (-1.03, 0.19) | (-1.34, 0.92) | (-1.19, 0.56) | (-1.02, 0.81) | 0.286 |
| **Feasibility** |  |  |  |  |  |  |  |  |  |  |  |
| Creation of an Interdisciplinary Committee | 4.20 (0.78) | 3.80 (0.94) | 4.06 (1.03) | 3.98 (0.85) | (-0.34, 1.03) | (-0.90, 0.60) | (-0.26, 0.69) | (-1.39, 0.39) | (-0.81, 0.54) | (-0.36, 1.09) | 0.367 |
| Establishing a mandatory One Health review system by institutions | 3.48 (1.11) | 3.29 (1.27) | 3.41 (1.12) | 3.27 (0.97) | (-0.38, 1.34) | (-0.75, 1.09) | (-0.25, 0.95) | (-1.41, 0.80) | (-0.98, 0.73) | (-0.72, 1.07) | 0.788 |
| Creation/use of Standard Operating Procedures (SOPs) for One Health proposals | 3.88 (0.95) | 4.07 (0.73) | 4.06 (0.66) | 4.11 (0.78) | (-0.81, 0.54) | (-1.03, 0.42) | (-0.61, 0.32) | (-1.04, 0.71) | (-0.69, 0.66) | (-0.55, 0.86) | 0.537 |
| Required training for all Committee members/Regulatory Body members | 4.00 (0.92) | 4.15 (0.55) | 3.94 (0.97) | 4.02 (0.94) | (-0.66, 0.77) | (-0.47, 1.03) | (-0.35, 0.63) | (-0.70, 1.14) | (-0.63, 0.80) | (-0.87, 0.59) | 0.917 |
| Improved channels for communication between researchers and reviewers/Regulatory Body members | 4.06 (0.68) | 3.93 (0.83) | 4.24 (0.90) | 3.91 (0.86) | (-0.60, 0.71) | (-1.02, 0.39) | (-0.30, 0.62) | (-1.23, 0.48) | (-0.56, 0.77) | (-0.21, 1.17) | 0.490 |
| Incentivizing those who review One Health research | 3.15 (1.09) | 3.77 (0.73) | 3.29 (1.16) | 3.39 (1.08) | (-1.51, 0.33) | (-1.05, 0.88) | (-0.88, 0.37) | (-0.67, 1.68) | (-0.58, 1.26) | (-1.10, 0.76) | 0.297 |
