# Supplementary material for: One Health research ethics review processes in African countries: Challenges and opportunities
Source: One Health. 2024 Mar 22;18:100716. doi: 10.1016/j.onehlt.2024.100716 (PMC11247289; doi:10.1016/j.onehlt.2024.100716)
Supplement: Supplementary file 14 — Supplementary material 14: Strategies to improve reviews of One Health research by Researchers, Research Ethics Committee Members, and Regulators under emergency situations (e.g. disasters, pandemics, etc.). Scores are represented in a Likert scale from 1-5; (5 representing highest agreement) and means (SD) are presented for the “Professional Role” columns. P-values are obtained from multivariable mixed effect models (which include other demographic variables, namely age, education, country of origin, sex and experience) and represent statistical significance for at least one role. [file mmc14.docx]

**S14 Table**. Strategies to **improve** reviews of One Health research by Researchers, Research Ethics Committee Members, and Regulators under **emergency situations** (e.g. disasters, pandemics, etc.). Scores are represented in a Likert scale from 1-5; (5 representing highest agreement) and means (SD) are presented for the “Professional Role” columns. P-values are obtained from multivariable mixed effect models (which include other demographic variables, namely age, education, country of origin, sex and experience) and represent statistical significance for at least one role.

| **Strategy** | **One Health Researcher** | **REC Member** | **Regulator** | **Multiple Roles** | **95% Confidence Interval** | | | | | | **P-value** |
| --- | --- | --- | --- | --- | --- | --- | --- | --- | --- | --- | --- |
|  |  |  |  |  | **One Health Researcher vs REC Member** | **One Health Researcher vs Regulator** | **One Health Researcher vs Multiple Roles** | **REC Member vs Regulator** | **REC Member vs Multiple Roles** | **Regulator vs Multiple Roles** |  |
| **Importance** |  |  |  |  |  |  |  |  |  |  |  |
| Creation of an ad hoc review committee for the emergency | 4.19 (0.79) | 3.79 (0.89) | 3.56 (0.96) | 3.83 (1.04) | (-0.22, 1.26) | (-0.40, 1.21) | (-0.06, 0.95) | (-1.09, 0.86) | (-0.81, 0.66) | (-0.75, 0.83) | 0.047 |
| Creation/use of Standard Operating Procedures (SOPs) for One Health proposals in emergencies | 3.98 (0.95) | 4.00 (0.71) | 4.27 (0.70) | 4.07 (0.90) | (-0.59, 0.88) | (-0.93, 0.71) | (-0.43, 0.56) | (-1.24, 0.74) | (-0.81, 0.65) | (-0.63, 0.98) | 0.717 |
| Required training focused on emergency situations for all members | 3.88 (0.92) | 4.36 (0.63) | 4.12 (0.89) | 3.80 (0.90) | (-0.92, 0.44) | (-0.62, 0.86) | (-0.24, 0.69) | (-0.53, 1.25) | (-0.21, 1.14) | (-0.62, 0.84) | 0.110 |
| Improved channels for communication during emergencies between researchers and reviewers/Regulatory Body members | 4.06 (0.76) | 3.93 (0.62) | 4.06 (0.68) | 3.91 (0.83) | (-0.41, 0.81) | (-0.73, 0.60) | (-0.29, 0.56) | (-1.06, 0.53) | (-0.67, 0.55) | (-0.45, 0.86) | 0.735 |
| Incentivizing reviewers specifically for emergency situations | 3.41 (1.22) | 3.92 (0.49) | 3.50 (1.10) | 3.56 (1.14) | (-1.15, 0.55) | (-0.76, 1.04) | (-0.65, 0.49) | (-0.66, 1.54) | (-0.63, 1.07) | (-1.11, 0.66) | 0.421 |
|  |  |  |  |  |  |  |  |  |  |  |  |
| **Feasibility** |  |  |  |  |  |  |  |  |  |  |  |
| Creation of an ad hoc review committee for the emergency | 4.14 (0.87) | 3.60 (1.12) | 3.79 (1.19) | 3.85 (0.97) | (-0.11, 1.43) | (-0.91, 0.95) | (-0.13, 0.93) | (-1.73, 0.45) | (-1.02, 0.50) | (-0.54, 1.30) | 0.181 |
| Creation/use of Standard Operating Procedures (SOPs) for One Health proposals in emergencies | 3.98 (0.88) | 3.79 (0.80) | 4.00 (0.78) | 4.04 (0.85) | (-0.47, 0.96) | (-0.91, 0.80) | (-0.46, 0.53) | (-1.30, 0.71) | (-0.92, 0.51) | (-0.74, 0.93) | 0.806 |
| Required training focused on emergency situations for all members | 3.82 (1.02) | 3.93 (0.70) | 3.64 (1.22) | 3.71 (0.89) | (-0.64, 0.86) | (-0.42, 1.39) | (-0.25, 0.81) | (-0.69, 1.43) | (-0.58, 0.91) | (-1.10, 0.69) | 0.784 |
| Improved channels for communication during emergencies between researchers and reviewers/Regulatory Body members | 4.10 (0.79) | 3.71 (1.07) | 3.64 (1.15) | 3.84 (0.85) | (-0.44, 1.08) | (-0.53, 1.25) | (-0.25, 0.77) | (-1.03, 1.10) | (-0.81, 0.69) | (-0.98, 0.78) | 0.232 |
| Incentivizing reviewers specifically for emergency situations | 3.44 (1.15) | 3.79 (0.70) | 3.29 (1.38) | 3.34 (1.14) | (-1.20, 0.67) | (-0.82, 1.37) | (-0.49, 0.80) | (-0.77, 1.84) | (-0.51, 1.34) | (-1.20, 0.96) | 0.579 |
|  |  |  |  |  |  |  |  |  |  |  |  |
