# Supplementary material for: One Health research ethics review processes in African countries: Challenges and opportunities
Source: One Health. 2024 Mar 22;18:100716. doi: 10.1016/j.onehlt.2024.100716 (PMC11247289; doi:10.1016/j.onehlt.2024.100716)
Supplement: Supplementary file 15 [file mmc15.docx]

OHEARP Survey

Start of Block: Introduction

Q1 

***The Ohio State University Consent to Participate in Research***

***Introduction***
*We are currently surveying One Health researchers, Research Ethics Committee (or Institutional Review Board) members, and members of Regulatory Bodies, with a focus on One Health research conducted in African countries.* 
For this survey, we are using the following definition: "One Health is a collaborative, multisectoral, and transdisciplinary approach—working at the local, regional, national, and global levels—with the goal of achieving optimal health outcomes recognizing the interconnection between people, animals, plants, and their shared environment."
https://www.cdc.gov/onehealth/index.html

 
***Purpose of the study*** 

*The objective of the study is to better understand the ethical challenges and opportunities in reviewing and conducting One Health research. Information from this survey will be used to make recommendations on the approval processes for One Health research, enabling important research to occur in a timely matter.* 

Q2 **Description of the Study Process**

If you consent to participate in the study, you will be directed to a 15-20 minute survey. 

**Participation Details:** 

Participation is voluntary. If you do not wish to take the survey, please click the "I do not consent" button below. Even if you consent to participate, you may leave the survey at any point. Partially completed responses will be used in analyses.

**Confidentiality:** 

Individual participant data is completely anonymous with no personal identifiers. The survey will not collect any institution/organization-related data or names. Only aggregated survey data will be used in the dissemination of research findings. 

In regards to internet confidentiality, we will work to make sure that no one sees your survey responses. However, since we are using the internet to administer the survey, there is a chance that someone could access your online responses and that this information could be used to identify your computer. Your de-identified information (all information provided in the survey) may be used or shared with other researchers without your additional informed consent.

**Risks, Benefits and Payment:** 

There are no individual benefits or risks associated with this research beyond the valuable information that will be gleaned from the survey itself. If you choose not to participate in this study, there are no penalties or negative consequences. There is no payment or incentives offered for participation in this survey.

Q3 **Questions:** 

If you have any questions regarding this survey, you may contact: - Dr. Donal O'Mathuna at omathuna.6@osu.edu; or - Dr. Getnet Yemir, at ali.578@osu.edu

For questions about your rights as a participant in this study or to discuss other study-related concerns, you may contact the OSU Office of Responsible Research Practices at +1-800-678-6251 or hsconcerns@osu.edu. 

Q4 **Consent**   

By selecting the "I consent to participate in this survey" button below, you indicate that you are willing to participate in this study, and that you have read and understood the information provided above. 

 

- I consent to participate in this survey (you will be directed to the survey immediately after selecting this response)  (1) 

- I do not consent to participate in this survey (you will not be directed to the survey, and no data will be collected)  (2) 

End of Block: Introduction

Start of Block: Demographics

Q5 For this survey, we are using the following definition: "**One Health** is a collaborative, multisectoral, and transdisciplinary approach—working at the local, regional, national, and global levels—with the goal of achieving optimal health outcomes recognizing the interconnection between people, animals, plants, and their shared environment."

https://www.cdc.gov/onehealth/index.html

Q6 Please identify your sex.

- Male  (1) 

- Female  (2) 

- Prefer not to say  (4) 

Q7 What is your age?

- 18-24 years old  (1) 

- 25-34 years old  (2) 

- 35-44 years old  (3) 

- 45-54 years old  (4) 

- 55-64 years old  (5) 

- 65-74 years old  (6) 

- 75 years or older  (7) 

Q8 Please identify your highest level of education completed. Please identify your degree, specialization or sub-specialization in the text box provided. Select all that apply. 

- High School Diploma (e.g. GED, Secondary Education Certificate, International Baccalaureate, or equivalent)  (1) 

- College/University Degree (e.g. BA, BSc)  (2) ________________________________________________

- Master's degree (e.g. MA, MSc, MBA, MBBS, MPH)  (3) ________________________________________________

- Doctorate (e.g. PhD)  (4) ________________________________________________

- Professional degree (e.g. DVM, MD) with specialization  (5) ________________________________________________

Q9 Are you a One Health researcher?

- Yes  (1) 

- No  (2) 

Display This Question:

If Q9 = 1

Q10 Please identify what best describes your current position. Select all that apply. 

- Assistant, Associate, or Full  Professor  (1) 

- Research Assistant  (2) 

- Graduate Student or Post-doctoral Fellow  (3) 

- Other (please specify):  (4) ________________________________________________

Display This Question:

If Q9 = 1

Q11 How long have you been in this position? (in years) 

- Less than 1  (1) 

- 1-5  (2) 

- 6-10  (5) 

- 11-19  (3) 

- 20+  (4) 

Q12 Do you serve on one or more Research Ethics Committees (also known as Institutional Review Boards/IRBs) ? 

- Yes  (1) 

- No  (2) 

Display This Question:

If Q12 = 1

Q13 Please identify the option which best describes your Committee. Select all that apply if you are on more than one Research Ethics Committee or IRB. 

- I serve on a Committee where One Health research is the primary or sole focus  (1) 

- I serve on a Committee where One Health research is reviewed occasionally or never  (2) 

- Other (please specify):  (4) ________________________________________________

Display This Question:

If Q12 = 1

Q14 How long (in years) have you been on this Committee? If you are on more than one Ethics Committee, answer for the Committee you have been on the longest. 

- Less than 1  (1) 

- 1-5  (2) 

- 6-10  (5) 

- 11-19  (3) 

- 20+  (4) 

Q15 Do you serve on a Regulatory Body (e.g. Ministry of Health, Ministry of Agriculture, Ministry of Environment, etc.)?

- Yes  (1) 

- No  (2) 

Display This Question:

If Q15 = 1

Q16 Please identify the option which best describes the Regulatory Body you serve on. 

- I am a member of a Regulatory Body with responsibilities for One Health research.  (1) 

- I am a member of a Regulatory Body with occasional or no responsibilities for One Health research.  (2) 

- Other  (3) ________________________________________________

Display This Question:

If Q15 = 1

Q17 How long (in years) have you been with this Regulatory Body? 

- Less than 1  (1) 

- 1-5  (2) 

- 6-10  (5) 

- 11-19  (3) 

- 20+  (4) 

Q18 What area(s) or discipline(s) do you currently work within? Select all that apply. 

- Animal Health sciences  (1) 

- Human Health sciences  (3) 

- Earth and Environmental sciences  (2) 

- Humanities and Social sciences  (4) 

- Natural Sciences  (5) 

- One Health sciences  (6) 

- Other (please specify):  (7) ________________________________________________

|  |
| --- |

Q19 In which country do you currently work? 

▼ Please select a country from the dropdown menu. (1360) ... Zimbabwe (186)

End of Block: Demographics

Start of Block: Research Ethics Committee Member

Q20 
The following questions are directed to participants with a role in Research Ethics Committees or Institutional Review Boards (IRBs). If you serve on more than 1 Committee, answer the questions for the Committee with the broadest engagement (e.g. for a national committee over a local committee).

For this survey, we are using the following definition: "One Health is a collaborative, multisectoral, and transdisciplinary approach—working at the local, regional, national, and global levels—with the goal of achieving optimal health outcomes recognizing the interconnection between people, animals, plants, and their shared environment."
https://www.cdc.gov/onehealth/index.html Or  

Q21 Which incentives do you have for serving on a Research Ethics Committee? Select all that apply. 

- Payment honorarium (e.g. for time spent)  (1) 

- Reimbursement of expenses (e.g. fuel costs)  (2) 

- Knowledge gain  (3) 

- Belief in the importance of ethical oversight of research  (8) 

- Personal satisifaction  (4) 

- Improving networking/collaboration opportunities  (5) 

- None  (7) 

- Other (please specify):  (6) ________________________________________________

Q22 What type of training in research ethics have you received? Select all that apply.

- Short-term formal training  - Training provided within your education or organization, with assessment of learning. Examples: Certificate program  (1) 

- Long-term formal training  - Training provided within your education or organization, with assessment of learning. Examples: Degree in Ethics/Bioethics or equivalent  (5) 

- Informal training - "On the job", learning through experience  (2) 

- Nonformal training - Training outside your organization that you found yourself, may or may not have assessment  (3) 

- Other (please specify):  (4) ________________________________________________

Q23 Is your Research Ethics Committee/IRB accredited? If Yes, please write in the name of the accreditation body. 

- Yes  (1) ________________________________________________

- No  (2) 

Q24 Does the Committee require all members to have formal training in research ethics (trainings with learning assessment)?

- Yes  (1) 

- No  (2) 

Q25 Does the Committee require a lay person/community member to participate in the review of One Health research proposals?

- Yes  (1) 

- No  (2) 

- Depends on the proposal  (3) 

Q26 Does your Committee use external One Health experts to help review One Health proposals? If yes, are they compensated? 

- No, we do not use external experts.  (1) 

- Yes, we use external experts and Yes, they are compensated for their time.  (2) 

- Yes, we use external experts, but No, they are not compensated for their time.  (3) 

- Yes, we use external experts, but I am unsure if they are compensated.  (4) 

Skip To: Q28 If Q26 = 1

Q27 How important are each of these challenges with finding external One Health experts or members with One Health expertise to serve on Research Ethics Committees? Please use the scale below to evaluate each proposed challenge

|  | Not at all important (14) | Slightly important (15) | Moderately important (16) | Very important (17) | Extremely important (18) |
| --- | --- | --- | --- | --- | --- |
| Lack of financial resources (1) |  |  |  |  |  |
| Lack of time (3) |  |  |  |  |  |
| Conflict of interest (e.g. experts often associated with the proposals) (2) |  |  |  |  |  |
| The expert's institution does not permit participation (20) |  |  |  |  |  |
| Other (please specify): (5) |  |  |  |  |  |

Q28 What is the most important challenge, in your opinion, in finding external experts or members with One Health expertise to serve on Research Ethics Committees? 

- Lack of financial resources  (1) 

- Lack of time  (3) 

- Conflict of interest (e.g. experts often associated with the proposals)  (2) 

- The expert's institution does not permit participation  (5) 

- Other (please specify):  (4) ________________________________________________

Q29 How important are each of these challenges in providing timely and appropriate review of One Health research? Please use the scale below to evaluate each proposed challenge.

|  | Not at all important (14) | Slightly important (15) | Moderately important (16) | Very important (17) | Extremely important (18) |
| --- | --- | --- | --- | --- | --- |
| Insufficient knowledge of One Health research (1) |  |  |  |  |  |
| Insufficient knowledge of ethical issues specific to One Health research (4) |  |  |  |  |  |
| Insufficient protocols for emergency situations (7) |  |  |  |  |  |
| Inadequate guidelines, protocols, SOPs (2) |  |  |  |  |  |
| Lack of a multidisciplinary review board (5) |  |  |  |  |  |
| Insufficient time for review (6) |  |  |  |  |  |
| Large number of proposals (8) |  |  |  |  |  |
| Other (please specify): (3) |  |  |  |  |  |

Q30 What is the most important challenge, in your opinion, in timely and appropriate review of One Health research? 

- Insufficient knowledge of One Health research  (1) 

- Insufficient knowledge of ethical issues specific to One Health research  (2) 

- Insufficient protocols for emergency situations  (7) 

- Inadequate guidelines, resources or SOPs  (3) 

- Lack of a multidisciplinary review board  (4) 

- Insufficient time for review  (5) 

- Other (please specify):  (6) ________________________________________________

Q31 Did your Committee provide training to assist you in reviewing specifically One Health research proposals?

- No, but I think I have adequate training  (1) 

- No, and I would like more training  (4) 

- Yes, but I would like more training  (2) 

- Yes, and it was adequate  (3) 

Q32 Does your Committee have specific standard operating procedures (SOPs) for One Health research proposals? This includes any written guidelines specific to One Health research.

- Yes  (1) 

- No  (2) 

- Unsure  (3) 

Display This Question:

If Q32 = 1

Q33 If yes, are the One Health research SOPs applied and used regularly by your Committee?

- Yes  (1) 

- No  (2) 

- Unsure  (3) 

Q34 From your experience as a Research Ethics Committee member, what is the average length of time (in months) that it takes for a One Health research proposal to be fully reviewed (approved or rejected)?

- Less than 1 month  (1) 

- 1-3 months  (2) 

- 4-5 months  (3) 

- 6 or more months  (4) 

Q35 Please provide an estimate of what percentage of proposals reviewed by the Committee you serve on are related to One Health research specifically.

- Less than 1%  (1) 

- 2-5%  (6) 

- 6-20%  (2) 

- More than 20 %  (3) 

Q36 Are there Standard Operating Procedures (SOPs) for review of One Health research proposals specifically for emergency situations (e.g. disasters, pandemics, etc.)?

- Yes  (1) 

- No  (2) 

Q37 What opportunities exist to make improvements in reviewing One Health research proposals as a Research Ethics Committee member? 

________________________________________________________________

________________________________________________________________

________________________________________________________________

________________________________________________________________

________________________________________________________________

End of Block: Research Ethics Committee Member

Start of Block: One Health Researcher

Q38 The following questions are directed to participants who are involved as researchers conducting One Health research.

For this survey, we are using the following definition: "One Health is a collaborative, multisectoral, and transdisciplinary approach—working at the local, regional, national, and global levels—with the goal of achieving optimal health outcomes recognizing the interconnection between people, animals, plants, and their shared environment."
https://www.cdc.gov/onehealth/index.html

|  |
| --- |

Q39 Which country(ies) are currently the focus for your One Health research collaborations? (Select all that apply. Hold the control ('Ctrl') key to select multiple countries.) 

- Ethiopia  (58) 

- Kenya  (89) 

- Tanzania  (3) 

- United States of America  (187) 

- Algeria  (3) 

- Angola  (5) 

- Benin  (19) 

- Botswana  (23) 

- Burkina Faso  (27) 

- Burundi  (28) 

- Cabo/Cape Verde  (1358) 

- Cameroon  (30) 

- Central African Republic  (33) 

- Chad  (34) 

- Comoros  (38) 

- Congo, Republic of the- Brazzaville  (39) 

- Côte d'Ivoire  (41) 

- Democratic Republic of the Congo  (47) 

- Djibouti  (49) 

- Egypt  (54) 

- Equatorial Guinea  (56) 

- Eritrea  (57) 

- Eswatini  (60) 

- Gabon  (66) 

- Gambia, The  (67) 

- Ghana  (70) 

- Guinea  (74) 

- Guinea - Bissau  (75) 

- Lesotho  (99) 

- Liberia  (101) 

- Libya  (100) 

- Madagascar  (104) 

- Malawai  (105) 

- Mali  (108) 

- Mauritania  (111) 

- Mauritius  (112) 

- Morocco  (119) 

- Mozambique  (120) 

- Nambia  (122) 

- Niger  (128) 

- Nigeria  (129) 

- Rwanda  (145) 

- Senegal  (153) 

- Seychelles  (155) 

- Sierra Leone  (156) 

- Somalia  (161) 

- South Africa  (162) 

- Sudan  (165) 

- Sudan, South  (166) 

- Togo  (175) 

- Tunisia  (174) 

- Uganda  (182) 

- Zambia  (1357) 

- Zimbabwe  (186) 

Q40 Approximately how many One Health-related proposals do you submit for ethical or regulatory approval each year? 

- 0  (4) 

- 1  (1) 

- 2-3  (2) 

- 4+  (3) 

Q41 Approximately how many different Regulatory Bodies and/or Ethics Committees must approve a One Health research project before it can start? (please provide your best estimate of an average number)

________________________________________________________________

Q42 From your experience, what is the average length of time (in months) that it takes for a One Health research proposal to be fully reviewed by all Regulatory Bodies and Ethics Committees required (and either approved or not approved)?

- Less than 1 month  (1) 

- 1-3 months  (2) 

- 4-5 months  (3) 

- 6 or more months  (4) 

Q43 Are there national* guidelines addressing the ethical conduct of research with human subjects (NOT LIMITED TO ONE HEALTH) in the countries where you conduct One Health research? Select all that apply. 

*Below your response(s), please identify the country(ies) you are referring to.

- Yes  (1) ________________________________________________

- No  (2) ________________________________________________

- Unsure  (3) ________________________________________________

Q44 Are there national* guidelines or regulations regarding the ethical conduct SPECIFICALLY of One Health research in the countries where you conduct One Health research? Select all that apply. 
*Below your response(s), please identify the country(ies) you are referring to.

- Yes  (1) ________________________________________________

- No  (2) ________________________________________________

- Unsure  (3) ________________________________________________

Q45 If you had a question about an ethical issue in One Health research, where would you go to get help addressing it? 

- Colleagues  (5) 

- Bioethics expert  (6) 

- Animal welfare expert  (8) 

- Reference resources (literature, website, manuals, published journals etc.)  (7) 

- Research Ethics Committee(s)  (2) 

- Regulatory Body/Governmental agency (health, environmental, etc.)  (3) 

- Other (please specify):  (4) ________________________________________________

Q46 How important are each of these challenges in receiving timely and appropriate review of One Health research? Please use the scale below to evaluate each proposed challenge.

|  | Not at all important (14) | Slightly important (15) | Moderately important (16) | Very important (17) | Extremely important (18) |
| --- | --- | --- | --- | --- | --- |
| Insufficient knowledge of One Health research in the review body or committee (1) |  |  |  |  |  |
| Insufficient knowledge of ethical issues specific to One Health research (4) |  |  |  |  |  |
| Insufficient protocols for emergency situations (2) |  |  |  |  |  |
| Lack of a multidisciplinary review board (5) |  |  |  |  |  |
| Insufficient time for review (6) |  |  |  |  |  |
| Other (please specify): (3) |  |  |  |  |  |

Q47 What is the most important challenge, in your opinion, in timely and appropriate approval of One Health research? 

- Insufficient knowledge of One Health research in the review body or committee  (1) 

- Insufficient knowledge of ethical issues specific to One Health research  (2) 

- Insufficient protocols for emergency situations  (3) 

- Lack of a multidisciplinary review board  (4) 

- Insufficient time for review  (5) 

- Other (please specify):  (6) ________________________________________________

Q48 Describe any ethics-related issues you encounter during the conduct of One Health research and how you address them. Provide as many examples as you wish. 

________________________________________________________________

________________________________________________________________

________________________________________________________________

________________________________________________________________

________________________________________________________________

Q49 What opportunities exist to make improvements in reviewing One Health research?

________________________________________________________________

________________________________________________________________

________________________________________________________________

________________________________________________________________

________________________________________________________________

End of Block: One Health Researcher

Start of Block: Regulatory Bodies 

Q50 
The following questions are directed to participants who have a role in Regulatory Bodies.

For this survey, we are using the following definition: "One Health is a collaborative, multisectoral, and transdisciplinary approach—working at the local, regional, national, and global levels—with the goal of achieving optimal health outcomes recognizing the interconnection between people, animals, plants, and their shared environment."
https://www.cdc.gov/onehealth/index.html

Q51 Have you ever had the opportunity to have training in the One Health approach?

- Yes  (1) 

- No  (2) 

Q52 What type of training in research ethics have you received? Select all that apply from the options below.

- Short- term formal training  - Training provided within your education or organization, with assessment of learning. Examples: Certificate program  (1) 

- Long- term formal training  - Training provided within your education or organization, with assessment of learning. Examples: Degree in Ethics/Bioethics or equivalent  (5) 

- Informal training - "On the job", learning through experience  (2) 

- Nonformal training - Training outside your organization that you found yourself, may or may not have assessment  (3) 

- None  (6) 

- Other (please specify):  (4) ________________________________________________

Q53 Did your Regulatory Body provide training to assist you in reviewing specifically One Health research proposals?

- No, but I think I have adequate training  (1) 

- No, and I would like to have more training  (4) 

- Yes, but I would like more training  (2) 

- Yes, and it was adequate  (3) 

Q54 Does your Regulatory Body use external One Health experts to help reviewing these types of proposals? If yes, are they compensated?

- No, we do not use external experts.  (1) 

- Yes, we use external experts and Yes, they are compensated for their time.  (2) 

- Yes, we use external experts, but No, they are not compensated for their time.  (3) 

- Yes, we use external experts, but I am unsure if they are compensated.  (4) 

Q55 How important are each of these challenges in addressing One Health research by your Regulatory Body? Please use the scale below to evaluate each proposed challenge.

|  | Not at all important (15) | Slightly important (16) | Moderately important (17) | Very important (18) | Extremely important (19) |
| --- | --- | --- | --- | --- | --- |
| Lack of time (3) |  |  |  |  |  |
| Lack of One Health research expertise (1) |  |  |  |  |  |
| Lack of research ethics expertise (24) |  |  |  |  |  |
| Inadequate guidelines, resources or SOPs (4) |  |  |  |  |  |
| Lack of legal framework (18) |  |  |  |  |  |
| Other (please specify): (5) |  |  |  |  |  |

Q56 What is the most important challenge, in your opinion, in addressing One Health research by your Regulatory Body? 

- Lack of time  (3) 

- Lack of One Health research expertise  (1) 

- Lack of research ethics expertise  (18) 

- Inadequate guidelines, resources or SOPs  (4) 

- Lack of legal framework  (2) 

- Other (please specify):  (5) ________________________________________________

Q57 Does your Regulatory Body have specific standard operating procedures (SOPs) for One Health research proposals?

- Yes  (1) 

- No  (2) 

Q58 From your experience as a Regulatory Body member, what is the average length of time (in months) that it takes for a One Health proposal to be fully reviewed (approved or rejected) by your Body?

- Less than 1 month  (1) 

- 1-3 months  (2) 

- 4-5 months  (3) 

- 6 or more months  (4) 

Q59 Please provide an estimate on what percentage of proposals reviewed by your Regulatory Body are related to One Health research specifically. 

- Less than 1%  (1) 

- 6-20%  (2) 

- More than 20%  (3) 

Q60 Are there Standard Operating Procedures (SOPs) regarding review of One Health research proposals specifically in emergency situations (e.g. disasters, pandemics)?

- Yes  (1) 

- No  (2) 

Q61 What opportunities exist to make improvements in reviewing One Health research as a Regulatory Body member? 

________________________________________________________________

________________________________________________________________

________________________________________________________________

________________________________________________________________

________________________________________________________________

End of Block: Regulatory Bodies 

Start of Block: Collaboration

Q62 In an ideal situation, how could reviews of One Health research by Research Ethics Committees and Regulatory Bodies be improved? Please use the scale below to evaluate each potential improvement method.

|  | Not at all important (14) | Slightly important (15) | Moderately important (16) | Very important (17) | Extremely important (18) |
| --- | --- | --- | --- | --- | --- |
| Creation of an Interdisciplinary Committee (5) |  |  |  |  |  |
| Establishing a mandatory One Health review system by institutions (10) |  |  |  |  |  |
| Creation/use of Standard Operating Procedures (SOPs) for One Health proposals (6) |  |  |  |  |  |
| Required training for all Committee members/Regulatory Body members (7) |  |  |  |  |  |
| Improved channels for communication between researchers and reviewers/Regulatory Body members (8) |  |  |  |  |  |
| Incentivizing those who review One Health research (11) |  |  |  |  |  |
| Other (please specify): (9) |  |  |  |  |  |

Q63 Given various restraints, how feasible (i.e. practically possible) would it be to make each potential change in the review of One Health research? Please use the scale below to evaluate each potential improvement method considering only their level of feasibility. 

|  | Not at all feasible (14) | Slightly feasible (15) | Moderately feasible (16) | Very feasible (17) | Extremely feasible (18) |
| --- | --- | --- | --- | --- | --- |
| Creation of an Interdisciplinary Committee (5) |  |  |  |  |  |
| Establishing a mandatory One Health review system by institutions (10) |  |  |  |  |  |
| Creation/use of Standard Operating Procedures (SOPs) for One Health proposals (6) |  |  |  |  |  |
| Required training for all Committee members/Regulatory Body members (7) |  |  |  |  |  |
| Improved channels for communication between researchers and reviewers/Regulatory Body members (8) |  |  |  |  |  |
| Incentivizing those who review One Health research (11) |  |  |  |  |  |
| Other (please specify): (9) |  |  |  |  |  |

Q64 Ideally, how could reviews of One Health research be improved during emergency situations (e.g. disasters, pandemics)? Please use the scale below to evaluate each potential improvement method. 

|  | Not at all important (14) | Slightly important (15) | Moderately important (16) | Very important (17) | Extremely important (18) |
| --- | --- | --- | --- | --- | --- |
| Creation of an ad hoc review committee for the emergency (5) |  |  |  |  |  |
| Creation/use of Standard Operating Procedures (SOPs) for One Health proposals in emergencies (6) |  |  |  |  |  |
| Required training focused on emergency situations for all members (7) |  |  |  |  |  |
| Improved channels for communication during emergencies between researchers and reviewers/Regulatory Body members (8) |  |  |  |  |  |
| Incentivizing reviewers specifically for emergency situations (11) |  |  |  |  |  |
| Other (9) |  |  |  |  |  |

Q65 Given various restraints, how feasible (i.e. practically possible) would it be to make each potential change in the review of One Health research during emergency situations (e.g. disasters, pandemics)? Please use the scale below to evaluate each potential improvement method considering only their level of feasibility. 

|  | Not at all feasible (14) | Slightly feasible (15) | Moderately feasible (16) | Very feasible (17) | Extremely feasible (18) |
| --- | --- | --- | --- | --- | --- |
| Creation of an ad hoc review committee for the emergency (5) |  |  |  |  |  |
| Creation/use of Standard Operating Procedures (SOPs) for One Health proposals in emergencies (6) |  |  |  |  |  |
| Required training focused on emergency situations for all members (7) |  |  |  |  |  |
| Improved channels for communication during emergencies between researchers and reviewers/Regulatory Body members (8) |  |  |  |  |  |
| Incentivizing reviewers specifically for emergency situations (11) |  |  |  |  |  |
| Other (9) |  |  |  |  |  |

End of Block: Collaboration

Start of Block: End

Q135 We will be conducting interviews later in 2021 on these topics. If you are interested in participating, please send an email to Dr. O' Mathuna at omathuna@osu.edu, so you can be added to our interview list. 

Q66 This is the end of the survey.

Thank you!

End of Block: End
